# The Salivary Microbiota–Host Nexus: Unraveling Opportunities for Non-Invasive Monitoring of Health and Productivity in Farm Animals

**DOI:** 10.3390/ani16121840

**Published:** 2026-06-15

**Authors:** Jing Ge, Kehui Ouyang, Mingren Qu, Qinghua Qiu

**Affiliations:** Jiangxi Province Key Laboratory of Animal Nutrition and Feed, College of Animal Science and Technology, Jiangxi Agricultural University, Nanchang 330045, China

**Keywords:** animal production, animal stress, biomarker, livestock health, non-invasive monitoring, saliva, salivary microbiota

## Abstract

Salivary microbiota constitutes complex microbial assemblages and acts as a source of reliable non-invasive biomarkers for evaluating growth, metabolism, and health status of farm animals. This review explores the research value of saliva and its resident microbes in livestock health monitoring. We summarize saliva composition, physiological functions, and sampling protocols for pigs, cattle, sheep, and goats. Core microbial taxa of monogastric and ruminant species are outlined, together with their roles in digestion, rumen fermentation, growth, and stress responses. We also present classic salivary diagnostic indicators and the impacts of oral bacteria on common livestock diseases. Current research is limited by undefined causal relationships, low diagnostic specificity, and heterogeneous technical standards, and thus fails to support accurate diagnosis at the individual animal level. Future studies should elucidate microbial interaction mechanisms, standardize experimental protocols, and establish multi-index evaluation models. This review advances theoretical research and promotes the practical application of salivary microbiota in precision livestock farming.

## Simple Summary

Saliva serves as a practical non-invasive medium for assessing animal health and physiological status. It contains abundant bioactive molecules and complex microbial communities, maintaining oral homeostasis and reflecting systemic physiological changes in livestock. This review explores the potential of salivary microbiota signatures as biomarkers for modern animal production. Shifts in microbial composition are associated with animal growth, production traits, and disease susceptibility. Analysis of salivary microbiota facilitates early disease screening, routine health surveillance, and better farm management. Most existing studies focus on community structure, whereas interactions between salivary microbes and host components remain poorly understood. Further research is required to clarify these interactions and validate the practical value of salivary biomarkers. Overall, salivary microbiota-based monitoring can effectively enhance animal health, welfare, and production efficiency in livestock farming.

## 1. Introduction

The oral cavity constitutes the anterior part of the digestive tract and also acts as an auxiliary respiratory passage for animals. It participates in core physiological activities such as feeding, mastication, swallowing, taste perception, and initial nutrient digestion [1]. Oral tissues are constantly immersed in saliva, a complex biofluid mainly secreted by major salivary glands. Saliva was once regarded solely as a digestive secretion that breaks down lipids and carbohydrates via endogenous enzymes [2]. Advances in oral microecology have greatly expanded our understanding of this fluid. A large number of studies confirm that saliva carries rich biomolecules and highly diverse microbial communities [3,4,5,6]. As a key part of the oral microecosystem, salivary microbiota has drawn growing research attention due to its close connection with the overall production performance of livestock.

Gut microbiota have long been regarded as important indicators for evaluating production traits in livestock husbandry [7]. Recent evidence shows salivary microbiota also correlate with animal growth and development, and take part in nutrient metabolism and physiological regulation. Bacteria in saliva help degrade plant fibers in the rumen of ruminants [8]. In vitro tests further prove that saliva and its microbes can modulate rumen microbial fermentation [9]. In addition, microorganisms adhering to oral structures and tissues, such as the oral mucosa and teeth, continuously disperse into and accumulate in saliva. This process gives the salivary microbiota a unique signature reflecting mucosal surface-associated oral microbiota [10], making saliva a suitable sample for screening disease markers and judging animal physical conditions.

Traditional approaches for assessing animal production performance and monitoring health status are often labor-intensive, invasive, time-consuming, or costly, which limits their ability to meet the increasing demand for real-time, non-invasive, and efficient biological information acquisition in livestock production. Although saliva is composed primarily of water, it is a complex mixture of proteins, electrolytes, and other organic compounds, mainly synthesized and secreted by the salivary glands, with a small proportion derived from the blood [11]. Material exchange between saliva and blood makes saliva an ideal medium to reflect host status and screen disease markers [12]. Saliva collection is simple and causes no harm to animals, and it supports repeated sampling across large herds [13]. Therefore, the use of salivary microbiota as a monitoring index in animal production systems may provide a promising strategy for improving production efficiency and animal health, while broadening the current focus beyond gut and fecal microbiota.

This review summarizes recent progress of salivary microbiota as a new non-invasive biomarker for livestock production. We first describe saliva composition and physiological functions, then analyze the characteristics and influencing factors of salivary microbiota in ruminants and monogastric animals, focusing on their effects on growth, production, and animal health. We also conclude existing research deficiencies and propose priorities for follow-up studies. This paper demonstrates the feasibility and broad application prospects of applying salivary microbiota to routine production monitoring in modern livestock farming.

## 2. Literature Search Strategy

**Figure 1:**
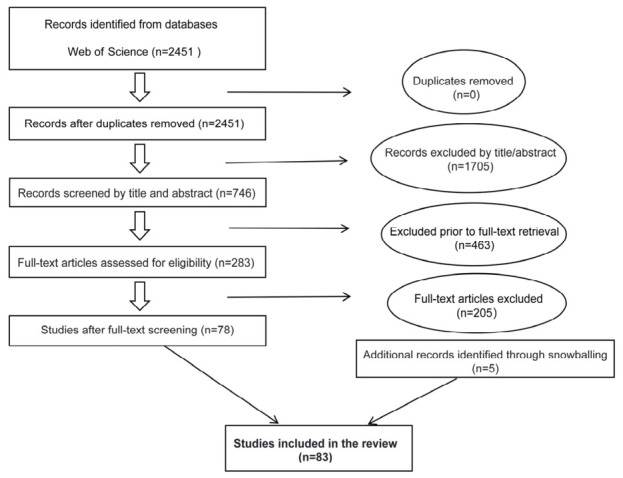
PRISMA flow diagram showing the screening process of eligible studies.

We searched peer-reviewed English articles published before April 2026 from Web of Science databases, focusing on salivary and oral microbiota related to ruminants and pigs. A standard Boolean search strategy was applied, with keywords combined as follows: (“saliva” OR “salivary microbiota” OR “oral microbiome” OR “salivary bacteria”) AND (“livestock” OR “ovine” OR “sheep” OR “goat” OR “cattle” OR “bovine” OR “pig”) AND (“health” OR “disease” OR “production” OR “performance” OR “diagnosis” OR “biomarker” OR “monitoring”). Duplicate records were removed first. We then screened papers by reading titles and abstracts to retain studies matching the research theme. Full-text evaluation was conducted for potentially eligible articles, and extra references were collected from the bibliography of included literature. All qualified papers formed the research basis of this review. The complete literature selection process is presented in a PRISMA-style flow chart (Figure 1).

## 3. Salivary Characteristics and Diagnostic Potential

### 3.1. Composition and Physiological Functions of Saliva

Saliva is a complex biofluid composed of salivary gland secretions, gingival crevicular fluid, exfoliated oral epithelial cells, and microorganisms [14]. It consists of approximately 99.5% water, with the remaining fraction comprising a range of solid components, including mucins, amylase, lysozyme, lactoferrin, immunoglobulins, and various electrolytes [1]. These substances work together to maintain oral environmental stability. Major electrolytes contain cations (e.g., Na^+^, K^+^, Ca^2+^, and Mg^2+^) and anions (e.g., HCO_3_^−^ and HPO_4_^2−^) [15]. Among these, bicarbonate and phosphate form the main buffering system, neutralizing acids produced by bacterial metabolism, maintaining oral pH within the optimal range of 6.5–7.5, and thereby providing a stable acid-base environment for microbial colonization and oral tissue function [16]. In addition, calcium and phosphate ions contribute to enamel remineralization and help reduce demineralization [17], which is important for protecting against tooth wear and dental caries in mammals, particularly ruminants exposed to prolonged mastication of high-fiber diets.

Beyond inorganic electrolytes, the organic fraction of saliva, although accounting for less than 1% of total salivary content, performs essential regulatory functions. The coordinated actions of inorganic and organic components are central to maintaining oral homeostasis, supporting normal microbial colonization, and preserving tissue integrity. As the major functional basis of salivary physiological activity, organic constituents can be broadly classified into four categories: proteins, enzymes, immunologically active molecules, and nitrogen-containing metabolites [18]. Collectively, these components support digestive tract health and performance through mucosal protection, initiation of digestion, immune defense, and microecological regulation.

Salivary enzymes carry out diverse biological roles. α-Amylase breaks down starch and starts carbohydrate digestion. Its activity is higher in monogastric animals fed starchy feed than in high-fiber-fed ruminants [19]. Lysozyme functions as an important antimicrobial enzyme by hydrolyzing bacterial cell walls and acts synergistically with other immune molecules [2]. Lingual lipase can resist acid and digest part of lipids inside the mouth and stomach. This enzyme matters greatly for young piglets and calves. Their pancreatic functions are not fully mature, and lingual lipase decomposes fat in milk to make up for insufficient pancreatic lipase. The resulting fatty acids can also promote pancreatic lipase secretion [11]. The source of salivary lipase still remains controversial. Human studies show it mainly comes from taste buds and oral microbes instead of major salivary glands [20], while relevant results differ between monogastric and ruminant livestock [21,22]. Large-scale systematic research is still needed to confirm its exact origin.

Secretory immunoglobulin A (sIgA) is a key immune component in saliva. It binds with mucosal mucins to build a protective layer, which prevents pathogenic bacteria and viruses from adhering to and invading epithelial cells, and lowers infection risks [23]. Produced by B cells in mucosal lymphoid tissues and secreted onto mucosal surfaces, sIgA interacts with other salivary ingredients to adjust oral microbial structure and maintain host health [24,25]. Lactoferrin restricts pathogen growth by sequestering iron ions and cutting off their nutrient supply [1]. Mucins trap pathogens with their viscosity. These microbes are swallowed and excreted, which helps keep oral microecology balanced [14].

Nitrogenous substances such as urea transfer from blood to salivary glands and finally enter saliva [26]. Their concentration changes reflect renal function and overall nitrogen metabolism. Impaired kidney function usually leads to an obvious rise in salivary urea content [15]. Salivary components have stable physical and chemical properties, which ensure reliable quantitative detection and practical diagnostic value [27]. Calice-Silva et al. [26] clarified the correlation between salivary nitrogen metabolites and human health, potentially providing ideas for applying saliva to non-invasive health monitoring using saliva in livestock production.

As an important carrier of the oral microecosystem, saliva achieves multiple physiological functions including oral protection, preliminary digestion, and microbial regulation. After being swallowed, it continues to work in the posterior digestive tract and affects animal growth and health. Mucins form a lubricating film on oral surfaces to reduce mechanical damage caused by coarse feed and prevent mucosal injury [1]. Buffering substances neutralize lactic acid produced by oral streptococci and protect tooth enamel from demineralization [17].

For ruminants, swallowed saliva regulates rumen acid-base balance and microbial fermentation, and further affects fiber degradation efficiency and nutrient digestibility [9,14,28]. Cattle and sheep show differences in this regulatory effect, largely due to distinct salivary composition and oral microbes between species [8,21]. Salivary amylase and lingual lipase assist carbohydrate and fat digestion for all farm animals [11,12]. Saliva secretion follows regular patterns closely linked to feeding behavior [29]. Feeding and rumination stimulate saliva production and enhance its buffering capacity, which in turn changes the rumen environment and microbial activity [30].

### 3.2. Methods of Saliva Collection and Detection

Saliva contains abundant physiological and microbial markers. Saliva testing can reflect an animal’s real status more truly than blood tests, and fully meets modern animal welfare requirements. Blood sampling easily causes wound infection and sample contamination in piglets, so saliva is a better choice for swine disease screening. Non-invasive samples including saliva, urine, and feces have gradually replaced blood for hormone and immune indicator detection, showing broad application prospects in pigs, sheep, and other livestock [31,32].

Sampling methods and tools directly affect animal stress and sample quality. Researchers need to select proper approaches according to actual production needs. Oral sponges and buccal swabs are widely used to collect saliva from cattle and sheep [33,34,35]. *Animals are generally fasted for 12 h before sampling with free access to clean water, to avoid contamination from rumen contents*. Oral sponges can collect enough samples for large-scale detection of microbes, metabolites, and physiological indicators. Samples are centrifuged and stored at low temperature immediately after collection [33,34]. *Researchers* rub buccal swabs gently on inner cheek surfaces for about 10 s to collect mucosal substances and oral microbes. This method fits the screening of oral flora and potential pathogens [33,35]. Pig saliva is mainly collected with hanging cotton ropes or sterile sponges. Researchers squeeze soaked cotton ropes or centrifuge used sponges to obtain saliva. The two methods apply to pigs at different growth stages. Cotton ropes work better for weaned piglets, while sterile sponges are more suitable for finishing pigs [36].

Standard sampling operations and contamination control are essential. Sampling time is another key factor influencing test results. Salivary microbes change obviously within a single day, and feed residues after eating can quickly alter microbial structure. Microbial composition and diversity also shift as animals grow and enter different physiological stages [37]. Inconsistent sampling time across studies contributes to inconsistent findings across studies. Low-temperature storage keeps enzymes and antibacterial substances in saliva active after collection. Saliva collection brings no physical harm to livestock. Secreted antibodies in saliva can also be used to evaluate host immune status.

Many technical factors may interfere with microbial detection results and reduce comparability between different studies. Different DNA extraction kits, sequencing platforms, and 16S rRNA gene primers will cause deviations in amplification and species identification. Various bioinformatics pipelines also change microbial classification results [38]. The lack of unified technical standards limits the repeatability and cross-study comparison of salivary microbiota research.

### 3.3. Classical Salivary Diagnostic Indicators and Applications

Saliva has complex components and unique physical properties. It maintains internal balance and acts as an excellent non-invasive diagnostic medium [31]. Changes in salivary ingredients directly reflect animal metabolism, immune level, stress state, and infection condition [39]. Mature diagnostic systems based on traditional salivary indicators have been established. Combined with microbial analysis, they realize multi-angle evaluation of livestock health and production performance.

Nitrogen-containing substances such as urea and creatinine in saliva have a strong correlation with blood biochemical indexes, and can be used to assess renal function and nitrogen metabolism. Studies prove these markers can stably reflect metabolic changes in ruminants under different feeding conditions, and serve as early warning signals for abnormal kidney function [40]. Salivary cortisol is a well-recognized stress marker widely used in animal production. Transportation, weaning, and heat stress will cause its content to fluctuate. Saliva collection does not bring extra stress to animals, so test results can truly reflect their stress level [41,42]. Acute-phase proteins including haptoglobin and adenosine deaminase are sensitive markers for inflammation. Their content rises sharply when animals suffer bacterial or viral infection and tissue damage, supporting early discovery of subclinical inflammation [42,43,44,45,46].

Saliva and oral fluid are suitable for large-scale herd screening of pathogens and specific antibodies. Test results agree well with blood detection, which helps carry out dynamic surveillance of infectious diseases on farms [47]. Combining traditional salivary indicators and microbiome research follows multi-omics ideas and helps explain the relationship between salivary components and host status. Classic salivary markers (e.g., nitrogen metabolites, stress hormones, acute-phase proteins, and pathogen antibodies) reflect metabolism, immunity, and inflammation, and are suitable for large-scale non-invasive group screening. Salivary microbial markers mainly show flora imbalance and excessive reproduction of opportunistic pathogens, to warn of hidden health risks and decreased production performance. The two detection methods complement each other. Combining biochemical indicators and microbial analysis achieves multi-dimensional health evaluation, with bright prospects in precision breeding and management based on multi-omics technology.

## 4. Association Between Salivary Microbiota and Host

### 4.1. Basic Composition of Salivary Microbiota

Oral microbes mainly live on the tongue surface, teeth, and gingival gaps. Their community structure updates constantly along with chewing, saliva flow, and swallowing [10]. Isolation culture, 16S rRNA sequencing and metagenomic sequencing are common methods to study host-related microbes. Human studies confirm healthy people have stable core oral flora [48,49]. Similar results are found in livestock. Healthy farm animals are mainly colonized by several core bacterial phyla, including *Firmicutes*, *Bacteroidota*, *Proteobacteria*, and *Actinobacteriota* [21,50,51]. The abundance and functions of these microbes differ by animal species and physiological state. Different microbial groups interact with each other, decompose nutrients and block pathogen invasion to maintain oral balance [3]. Microbial structure will change when animals get sick, so salivary microbes have great potential as disease diagnostic markers [52].

Animal species determines the overall structure of salivary microbial communities. Pig saliva is dominated by *Firmicutes* and *Proteobacteria*, with *Streptococcus* and *Lactobacillus* as the main genera [21]. For cattle and sheep, *Proteobacteria* and *Actinobacteriota* take the dominant position. Their oral microbes differ greatly from rumen microbes, where *Prevotella* is abundant. This shows obvious niche distribution characteristics of digestive tract flora [51,53]. Animal growth also changes oral flora. Young animals have unstable microbial communities. Their flora gradually becomes stable as body functions mature, and the quantity of core microbes changes regularly with age [54]. Rearing conditions also exert an obvious influence on salivary microbiota. Feed formula, feeding mode, breeding density, and living environment will reshape oral microbial communities, and change microbial diversity and dominant species proportion. Animals raised under different management modes show distinct salivary microbial features [33,51].

Healthy livestock carry certain opportunistic pathogens in saliva normally, such as *Erysipelothrix*, *Actinobacillus*, and *Streptococcus* spp. These microbes stay at low abundance and cause no disease under normal conditions. High breeding density, feed switching, long-distance transport and decreased immunity will break oral balance and lead to massive reproduction of opportunistic pathogens [22,55,56]. Excessive *Erysipelothrix* may cause swine erysipelas, accompanied by systemic inflammation and reduced production performance [57]. Elevated oral *Actinobacillus* and *Streptococcus* abundances directly cause oral lesions, respiratory infections, growth retardation, and impaired welfare in intensively farmed animals [56,57,58]. *Streptococcus suis* enhances its colonization and pathogenic ability under environmental stress through transcription factor PrlP [59]. *Actinobacillus* tends to cause disease when oral mucosal barriers are damaged and animals live in crowded conditions [58].

### 4.2. Direct Driving Effect of Salivary Microbiota on Animal Growth Performance

Salivary microbiota plays an important role in nutrient decomposition, feed utilization, and animal growth. Core oral bacteria break down carbohydrates and fiber in feed and further affect nutrient absorption and host growth traits. *Streptococcus* and *Prevotella* are dominant in pig saliva [60]. Their abundance changes correlate with nutrient digestion efficiency [55]. Murase et al. [22] conducted microbial analysis encompassing oral, fecal, vaginal, and environmental samples, identifying pig saliva as the primary natural habitat for *Streptococcus suis*. This genus occupies a large proportion of pig saliva and keeps a stable dominant position. Buiatte et al. [21] studied oral microbes of nursery pigs, also including fecal and environmental controls. They found *Streptococcus* is the dominant genus in oral fluid, while *Prevotella* 9 is highly enriched in feces and also detectable in the oral cavity. As a member of the genus *Prevotella*, its abundance is related to digestion efficiency [61], potentially suggesting its function in polysaccharide degradation and nutrient utilization. Wang et al. [62] compared oropharyngeal microbes between healthy and diseased piglets, and further verified that *Streptococcus* and *Prevotella* form the core flora of healthy individuals. These microbes maintain normal nutrient metabolism in pig bodies.

For ruminants, microbes exchange between the oral cavity and rumen. Salivary microbes can change rumen flora structure and fermentation functions [51]. Fiber-degrading microbes from saliva enter the rumen and cooperate with local microbes to improve roughage utilization. Feed characteristics affect saliva secretion and microbial transmission during feeding [63]. Edwards et al. [8] confirmed salivary microbes are not just transient organisms in the rumen. They secrete fiber-degrading enzymes and assist rumen microbes in decomposing plant materials. This synergistic effect improves roughage utilization, especially for coarse forage. Palma-Hidalgo et al. [64] found that salivary ingredients can raise total volatile fatty acids (VFAs) and propionate content in the rumen. The two substances are key indicators of energy supply and growth status for ruminants. Recent studies show cattle oral microbes can reflect feed utilization and growth performance. Specific microbial groups in Prevotellaceae relate to feed conversion ratio and average daily gain, and can be used to screen high-efficiency individuals [61]. In general, salivary microbial composition and stability connect with feed utilization and production performance for both monogastric and ruminant animals.

### 4.3. The Critical Role of Salivary Microbiota in Animal Disease Prevention and Control

The oral cavity is the first place where animals contact external pathogens. Oral microbes form a protective barrier and regulate host immunity, becoming the first line of defense against oral and digestive tract infectious diseases. Oral microbes can also be used as non-invasive markers for early disease detection and risk assessment.

Many studies have confirmed the protective effect of salivary microbiota on animal respiratory health. Su et al. [65] reported that *Streptococcus salivarius* K12, a common oral commensal bacterium, confers protection against *Mycoplasma pneumoniae* infection via three mechanisms. It reduces pathogen quantity by inhibiting virulence factors such as P1 adhesion protein and community-acquired respiratory distress syndrome (CARDS) toxin. It suppresses the toll-like receptor 2 or toll-like receptor 4 (TLR2/TLR4) signaling pathway to cut the secretion of inflammatory factors, including tumor necrosis factor-alpha (TNF-α) and interleukin-6 (IL-6), and relieve excessive inflammation. It also prevents airway tissue damage and maintains complete respiratory barriers. These findings reveal the interaction between the mouth and the respiratory tract, and provide a new non-invasive method for livestock respiratory disease prevention.

High-grain feeding easily causes subacute ruminal acidosis (SARA) in ruminants. Concentrated feed inhibits saliva secretion, reduces rumen acid neutralization ability, changes salivary and rumen microbial composition, and disturbs normal rumen fermentation [66,67,68]. Traditional SARA diagnosis relies on invasive rumen fluid collection. Clinical symptoms usually appear late and are not obvious, which cannot meet production demands. Based on ruminant rumination characteristics, Liu et al. [69] applied buccal microbiota to SARA diagnosis. Buccal swab sampling is simple and causes little stress to animals. *Prevotellaceae UCG-003* can decompose fiber and assist in disease diagnosis at the same time.

*Streptococcus suis* infection is widespread in large-scale pig farms. Traditional diagnosis depends on blood collection and pathogen isolation. The process is time-consuming and not suitable for large-scale early screening. López-Martínez et al. [70] established a salivary indicator system for *Streptococcus suis* infection. Saliva can reflect physiological and pathological changes after infection. The quantity of *Streptococcus suis* and inflammatory factors such as IL-6 and TNF-*α* in saliva can be used as early warning markers [71]. This work fills the research gap of saliva application in swine bacterial disease monitoring, provides practical support for pig health management, and offers a reference for combined diagnosis with biochemical indicators. However, it should be noted that *Streptococcus suis* is a common commensal bacterium colonizing the mucosal surface of clinically healthy pigs. The pathogenicity of *Streptococcus suis* is highly strain-specific, and colonizing strains and invasive pathogenic strains differ significantly in genetic characteristics and virulence. Therefore, salivary *Streptococcus suis* abundance and inflammatory indicators can only serve as preliminary warning signals for herd health monitoring and cannot replace etiological identification or strain typing for individual diagnosis, which also complicates precise treatment and vaccination strategies.

Saliva detection also supports viral disease surveillance in livestock. Baumann et al. [72] established a new saliva sampling method for bovine respiratory virus detection. It suits large-scale herd monitoring, but limited sensitivity and accuracy restrict its use for single animal diagnosis. For swine salmonellosis, De Lucia et al. [73] analyzed antibody levels in saliva and blood of finishing pigs. The two groups show a moderate but significant correlation. Saliva antibody content is lower than that in blood, but saliva testing is still a practical non-invasive method for group epidemic monitoring. Individual differences and inconsistent results between saliva and blood mean this method only applies to herd screening, rather than a definite diagnosis for single infected animals.

**Figure 2:**
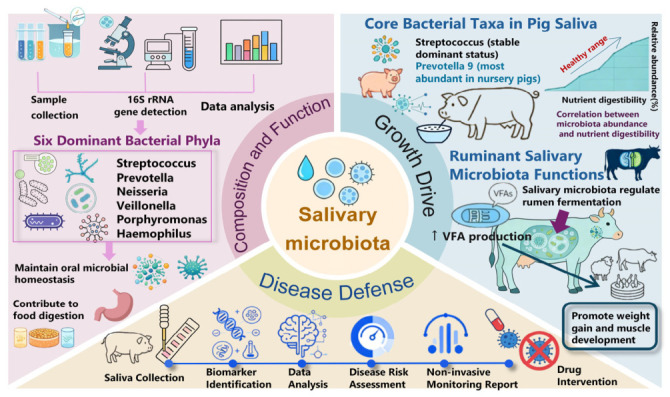
Functions of salivary microbiota. The upward arrow (↑) denotes increased VFAs production.

Collectively, the composition, growth-driving effects, and disease-defense functions of salivary microbiota in livestock are presented in Figure 2. To complement this overview, Table 1 further summarizes dominant salivary microbes, related production traits, associated diseases, and biomarker applications of major farm animals, offering a clear collation of existing research results.

## 5. Discussion and Future Perspectives

Salivary components exchange substances with blood through salivary gland epithelial barriers, so saliva composition changes can reflect the animal’s physiological state, metabolism, and disease conditions. Saliva is widely recognized as an important source of detection markers in modern livestock production [77]. Salivary microbiota has become a research hotspot in disease monitoring. Changes in microbial structure, inflammatory substances, and antibodies in saliva support early disease diagnosis. For example, changes in salivary *Streptococcus* combined with increased inflammatory markers have an obvious correlation with *Streptococcus suis* infection [22]. Saliva testing works well for group health monitoring, but it cannot distinguish different pathogen strains and cannot replace a definite diagnosis for individual animals.

Changes in *Prevotellaceae UCG-003* abundance in saliva relate to SARA in ruminants [69]. Salivary microbial markers can warn of group health risks and support routine monitoring under intensive farming conditions. Salivary microbiota has broad application prospects in disease prevention and non-invasive monitoring, while current research still has many deficiencies. Most studies only find a correlation between oral flora imbalance and diseases, but cannot confirm a causal relationship. It remains unclear whether microbial dysbiosis precedes disease onset, or if it is just a result of inflammation and metabolic disorder. Salivary markers also have low diagnostic specificity. Diet, growth stage, environment, and management can all change microbial structure. These changes are not unique to certain diseases, which limits practical application. The whole field lacks unified technical standards. Differences in sampling time, feed, animal age, detection kits, sequencing platforms, and analysis methods lead to inconsistent results, and poor repeatability and comparability between studies. Current saliva-based detection methods are mainly designed for group screening and epidemic warning. Large individual differences make accurate single-animal diagnosis difficult and hinder the translation of this technology into practical use. These shortcomings do not deny the advantages of salivary microbiota as a non-invasive research tool. Solving the above problems is the core direction of follow-up research.

Many basic scientific questions remain unanswered. It is still unclear whether ruminant salivary microbiota can replace rumen microbiota for routine sampling and detection [51]. Host status, microbial interaction, saliva pH, and diet jointly regulate oral flora structure and functions, while the specific mechanisms are not fully explained [78]. Salivary microbes change obviously under different physiological and feeding conditions [61,79,80], but the molecular causes behind these changes are still unknown. Most existing research adopts single-angle analysis. Some studies focus on microbial functions in digestion and health, while others explore non-microbial components in saliva. Few works discuss the combined effects of microbes and salivary proteins, hormones, and metabolites. Studies show sheep saliva can affect plant growth [75], but the joint role of salivary microbes and active substances is not clear. Diet and management change saliva secretion, composition, and rumen fermentation [81]. Disturbance of the saliva–rumen system develops over time in high-grain-fed dairy cows [82]. How salivary microbes change with feeding time and interact with saliva properties during rumen regulation still needs further exploration. These unknown points restrict the popularization of salivary microbiology in animal production.

Future research should move beyond single-dimensional analysis and build multi-dimensional research systems combining microbial, biochemical, and physiological indicators. Large-scale cross-species comparison is needed to solve key scientific problems, including interspecific differences in salivary lipase and the reliability of salivary microbes as rumen microbe substitutes. Combining multi-omics and molecular technology helps explain interaction mechanisms between salivary microbes and host components, and find key pathways related to nutrition, immunity and disease. For practical application, researchers need to build multi-index diagnosis models and formulate unified rules for saliva sampling and testing. Field verification on farms and the development of portable testing tools will accelerate the transformation of research achievements. In addition, screening and functional verification of beneficial salivary microbes can support the development of new microecological products to improve animal production performance and health [83].

## 6. Conclusions

Salivary microbiota can be collected through non-invasive methods. Its composition correlates with host metabolism, growth, and disease occurrence, and it has high application value in herd health monitoring and production evaluation. Current research encounters several key bottlenecks, including ambiguous causal relationships between microbial dysbiosis and disease occurrence, insufficient diagnostic specificity, lack of unified sampling and sequencing standards, poor result repeatability, and inability to achieve accurate individual-animal diagnosis. Unifying experimental specifications, exploring interaction mechanisms between salivary components and microbes, building multi-index diagnostic models, and carrying out on-site farm tests will further promote the application of salivary microbial markers in modern livestock health management and precision breeding.

## Figures and Tables

**Table 1:** Dominant salivary microbiota taxa, associated diseases, and biomarker applications in farm animals.

Animal Species	Dominant Salivary Taxa	Associated Production Traits	Associated Diseases	Biomarker Applications	References
Growing and nursery pigs	*Streptococcus*; *Prevotella* 9; *Actinobacillus*; *Erysipelothrix*	Improved nutrient digestibility and feed conversion; Promoted average daily gain; Enhanced oral polysaccharide degradation	Porcine respiratory disease; *Streptococcus suis* infection; Stress-induced growth retardation	Herd growth assessment; Early warning of bacterial infection and subclinical stress	[21,22,31,60,62]
Dairy and beef cattle	*Streptococcus*; *Actinomyces*; *Fusobacterium*; *Prevotella*	Increased roughage digestibility; Optimized rumen VFA composition; Improved milk yield and body weight gain	Subacute ruminal acidosis; Bovine respiratory disease; Rumen fermentation disorder	Non-invasive SARA diagnosis; Lactation and growth performance prediction	[8,35,64,69,74]
Sheep and goats	Lachnospiraceae; *Fibrobacter*; *Prevotellaceae UCG-003*; *Succinivibrio*; *Ruminococcus*; *Treponema*	High forage utilization and nutrient retention; Balanced rumen fermentation; Strong adaptability to coarse feed	Subacute ruminal acidosis; Rumen microecological imbalance; Nutritional metabolic disorders; Stress-restricted growth	Early SARA screening; Evaluation of rumen function and nutritional regulation	[28,64,69,75,76]

## Data Availability

No new data were created or analyzed in this study. Data sharing is not applicable to this article.

## Abbreviations

The following abbreviations are used in this manuscript:

sIgA	Secretory immunoglobulin A
SARA	Subacute ruminal acidosis
CARDS	Community-acquired respiratory distress syndrome
TLR2	Toll-like receptor 2
TLR4	Toll-like receptor 4
TNF-α	Tumor necrosis factor-alpha
IL-6	Interleukin-6
VFAs	Volatile fatty acids
